# Long-Term Outcomes in Puerto Ricans with Rheumatoid Arthritis (RA) Receiving Early Treatment with Disease-Modifying Anti-Rheumatic Drugs using the American College of Rheumatology Definition of Early RA

**DOI:** 10.2174/1874312901711010136

**Published:** 2017-12-27

**Authors:** Noemí Varela-Rosario, Mariangelí Arroyo-Ávila, Ruth M. Fred-Jiménez, Leyda M. Díaz-Correa, Naydi Pérez-Ríos, Noelia Rodríguez, Grissel Ríos, Luis M. Vilá

**Affiliations:** 1 Division of Rheumatology, Department of Medicine, University of Puerto Rico Medical Sciences Campus, San Juan, Puerto Rico; 2 Puerto Rico Clinical and Translational Research Center, University of Puerto Rico Medical Sciences Campus, San Juan, Puerto Rico

**Keywords:** Rheumatoid arthritis, Early Treatment, Disease-Modifying anti-rheumatic drugs, Clinical Outcome, Hispanics, Puerto Ricans

## Abstract

**Background:**

Early treatment of rheumatoid arthritis (RA) results in better long-term outcomes. However, the optimal therapeutic window has not been clearly established.

**Objective:**

To determine the clinical outcome of Puerto Ricans with RA receiving early treatment with conventional and/or biologic disease-modifying anti-rheumatic drugs (DMARDs) based on the American College of Rheumatology (ACR) definition of early RA.

**Methods:**

A cross-sectional study was performed in a cohort of Puerto Ricans with RA. Demographic features, clinical manifestations, disease activity, functional status, and pharmacotherapy were determined. Early treatment was defined as the initiation of DMARDs (conventional and/or biologic) in less than 6 months from the onset of symptoms attributable to RA. Patients who received early (< 6months) and late (≥6 months) treatments were compared using bivariate and multivariate analyses.

**Results:**

The cohort comprised 387 RA patients. The mean age at study visit was 56.0 years. The mean disease duration was 14.9 years and 337 (87.0%) patients were women. One hundred and twenty one (31.3%) patients received early treatment. In the multivariate analysis adjusted for age and sex, early treatment was associated with better functional status, lower probability of joint deformities, intra-articular injections and joint replacement surgeries, and lower scores in the physician’s assessments of global health, functional impairment and physical damage of patients.

**Conclusion:**

Using the ACR definition of early RA, this group of patients treated with DMARDs within 6 months of disease had better long-term outcomes with less physical damage and functional impairment.

## 1 INTRODUCTION

Suboptimal treatment of Rheumatoid Arthritis (RA) is associated with a greater risk of extra-articular manifestations, significant disability, and early mortality [1, 2]. Over the past decades, there has been an increased awareness of the beneficial impact of early detection and prompt treatment of RA with disease-modifying anti-rheumatic drugs (DMARDs) in order to retard disease progression and even attain clinical remission as a possible goal [3-6]. Most studies on early RA have been conducted in North America and Europe, and published data generally are based on patients receiving DMARDs within 12 or even 24 months after RA diagnosis [3-10]. However, very few outcome studies have been performed using the American College of Rheumatology (ACR) definition of early RA, which encompasses patients having disease duration of less than 6 months from the onset of symptoms attributable to RA [11]. Furthermore, there are few studies of early RA conducted in Hispanics, and to the best of our knowledge, there are no published data using the current ACR definition of early RA in this ethnic group. Thus, we sought to determine the clinical outcome in a cohort of Hispanics from Puerto Rico with RA treated with DMARDs within 6 months from the onset of symptoms attributable to RA and compare their outcome to those who received late treatment.

## 2 METHODS

### 2.1 Patient Population

Our cohort of RA patients has been described in detail before [12, 13]. In short, all patients met the 1987 ACR classification criteria for RA [14] and had Puerto Rican ethnicity. RA patients were enrolled at university-based (University of Puerto Rico Medical Sciences Campus San Juan, Puerto Rico) and community-based (San Juan, Puerto Rico) rheumatology clinics. Patients were recruited between February 2007 and June 2015. The study was approved by the Institutional Review Board of the University of Puerto Rico Medical Sciences Campus.

### 2.2 Variables

For the analyses, patients were categorized into two groups based on the time frame of initiation of DMARDs (conventional and/or biologic) treatment. Early treatment was defined as the initiation of DMARDs within 6 months from the onset of symptoms attributable to RA and late treatment as the start of DMARDs at 6 months or beyond the onset of RA symptoms.

As described before, our database comprises variables from the demographic, health-related behaviors, clinical, and pharmacologic domains [12, 13]. Demographic features and health-related behaviors included age (at study visit), sex, disease duration (time interval between RA onset and study visit), and current status (within the past 30 days) of cigarette smoking, alcohol consumption and exercise. Alcohol consumption was defined as ≥ 1 drink (0.6 ounces or 14.0 grams of pure alcohol) per day for women and ≥ 2 drinks per day for men. Exercise was defined as regular participation in physical activity as part of a health-enhancing personal fitness plan. Exercise activities included aerobic exercise and/or muscle strength training performed at least three times per week for 30–60 minutes per session.

Clinical variables including RA manifestations and cumulative comorbidities were determined at study visit. The following RA manifestations were ascertained: joint deformities and/or contractures, radiographic joint damage, positivity to rheumatoid factor and/or anti-citrullinated protein (CCP) antibodies, extra-articular manifestations (ocular, pulmonary, cardiac, neurologic, and subcutaneous nodules), intra-articular corticosteroid injections, joint replacement surgeries, RA exacerbations, hospitalizations, disease activity (per European League Against Rheumatism Disease Activity Score 28 (DAS28) [15]), clinical remission (DAS28 <2.6), functional status (per Health Assessment Questionnaire (HAQ) [16]), patient’s global assessment, and physician’s assessments of global health, functional impairment and physical damage of patients [17, 18].The following comorbid conditions were assessed: arterial hypertension, type 2 diabetes mellitus (DM), dyslipidemia, overweight and/or obesity (body mass index ≥ 25.0), cardiovascular events, cataracts, glaucoma, depression, malignancy, Sjögren’s syndrome, osteoarthritis, and osteoporosis.

The initial and cumulative exposure to corticosteroids, Nonsteroidal Anti-Inflammatory Drugs (NSAIDs), conventional DMARDs, biologic agents, tofacitinib, and different drug combinations were determined.

### 2.3 Statistical Analysis

Descriptive statistics were used to characterize the study group. Chi-squared test, Fisher’s exact test, or Student’s t tests, as appropriate, were performed to determine association of participants’ demographic features, clinical manifestations, comorbid conditions, and medications and time of treatment with conventional and/or biologic DMARDs (early vs. late). Age-and sex-adjusted odds ratios were estimated, along with 95% Confidence Intervals (CI), using a logistic regression analysis. Variables that showed statistical significance in the bivariate analyses were entered into the multivariable analyses. P-values ≤ 0.05 were considered statistically significant. All statistical analyses were performed using the Statistical Package STATA (StataCorp. 2015. Release 14. College Station, TX).

## 3 RESULTS

A total of 387 patients with RA were studied. At study visit, the mean (standard deviation [SD]) age of the study population was 56.0 (14.0) years. The mean (SD) disease duration was 14.9 (51.5) years and 337 (87.0%) patients were women. One hundred and twenty-one (31.3%) patients received early treatment and 266 (68.7%) received late treatment. Table **1** shows the demographic features and health-related behaviors in RA patients receiving early and late treatment with conventional and/or biologic DMARDs. Patients receiving early treatment were younger than those who received late therapy (53.6 vs. 57.1 years, p = 0.022). No differences were seen for sex or lifestyle behaviors.

Table **2** shows the clinical manifestations, disease activity and outcomes. RA patients who received early treatment were less likely to have joint deformities (35.5% vs. 55.3%, p<0.001) and subcutaneous nodules (19.0% vs. 29.7%, p=0.027), and require less intra-articular injections (40.0% vs. 54.5%, p=0.008) and joint replacement surgeries (9.2% vs. 22.6%, p=0.002) than those who received late treatment. They also had significantly lower mean scores in the HAQ (0.9 vs. 1.2, p<0.001), and physician’s assessments of global health (17.1 vs. 22.0, p=0.041), functional impairment (16.3 vs. 25.1, p<0.001) and physical damage (14.3 vs. 24.1, p<0.001). There were no significant differences in the disease duration, radiographic evidence of joint damage, rheumatoid factor or anti-CCP antibodies positivity, extra-articular manifestations besides subcutaneous nodules, RA exacerbations, hospitalizations, disease activity, clinical remission, or patient’s global health assessment.

Among comorbid conditions (Table **3**), type 2 DM was significantly more frequent in patients who received early treatment with DMARDs than in those who had late treatment (20.6% vs. 12.8%, p=0.046). No differences were noted for other comorbid conditions. The initial and cumulative pharmacologic RA therapies are described in Table **4**. No significant differences were observed for NSAIDs, corticosteroids, DMARDs (traditional and biologic), and drug combinations between the study groups.

In the multivariate analysis adjusted for age and sex, early treatment was associated with less joint deformities (odds ratio [OR] 0.49, 95% CI 0.31-0.79), intra-articular injections (OR 0.61, 95% CI 0.39-0.96) and joint replacement surgeries (OR 0.37, 95% CI 0.18-0.73), and lower scores in HAQ (OR 0.53, 95% CI 0.34-0.83), and physician’s assessments of global health (OR 0.56, 95% CI 0.36-0.88), functional impairment (OR 0.54, 95% CI, 0.34-0.86) and physical damage (OR 0.58, 95% CI 0.37-0.91). Conversely, early treatment was associated with type 2 DM (OR 2.13, 95% CI, 1.18-3.85). The association with subcutaneous nodules did not retain significance. These results are depicted in Table **5**.

## 4 DISCUSSION

The benefit of early RA treatment with DMARDs has been extensively studied over the last years and there is a consensus among rheumatologists for the need of prompt intervention to alter the potentially devastating disease course. However, different definitions to describe early disease have been used in clinical studies, ranging from 3 months to 2 years after the diagnosis of RA [3-10]. The current ACR guidelines for the treatment of RA define early disease as those patients having less than 6 months from the onset of symptoms attributable to RA [11]. Only a few studies have been conducted using this time frame. Thus, we determined the clinical outcome in a cohort of Puerto Ricans with RA receiving early treatment with DMARDs based on the ACR definition of early RA. We found that early treatment was associated with higher functional status, lower probability of joint deformities, intra-articular injections and joint replacement surgeries, and lower scores in the physician’s assessments of global health, functional impairment and physical damage when compared to those who received late treatment. Noteworthy, no significant differences were observed for the initial and cumulative exposure to specific conventional or biologic DMARDs among the study groups.

The findings of our study are in accordance with those observed in the ESPOIR cohort [19, 20]. The latter is a longitudinal prospective observational study of 813 initially DMARD-naïve patients from the French Early Arthritis Clinic followed since 2002. In this study, those who received treatment within 6 months of symptom onset had less joint deformities, intra-articular injections and joint replacement surgeries, and had better functional capacity. Some aspects of this study are comparable to ours. For example, in both cohorts the choice of DMARD therapy was left to the primary rheumatologist owing to the fact that no treatment protocol was established on either cohort; thus reflecting the daily practice in rheumatology. As per the 5-year data report of the ESPOIR cohort the use of conventional and biologic DMARDs was similar to that in our cohort [18]. MTX was the primary conventional DMARD of choice; however, compared with our study a higher use of sulfasalazine and leflunomide was observed. Conversely, hydroxychloroquine was used more frequently in our group. With regard to biologic DMARDs, TNF-α inhibitors were the most common biologic therapy used in both studies, but their use was higher in the ESPOIR group. Also, the exposure to corticosteroids was comparable as approximately 50% of the patients in both cohorts received treatment with these drugs at some point during the disease course. Although the studies differ in some aspects of the methodology, such as the ESPOIR is a prospective longitudinal study assessing not only clinical variables but also other factors such as HLA-DRB1 genotyping, synovial immune-histologic markers, radiographic progression, work disability, and economic impact of treatment and mortality, both reach similar conclusions regarding the benefits of early intervention in RA using the 6-month time frame [19, 20].

Few RA studies have been conducted in Latin America, and besides our work, none have compared the long-term outcomes of early versus late treatment [21-24]. Some of the demographic and clinical characteristics observed in our group are comparable with the Latin American cohorts GLADAR (Grupo Latino Americano de Estudio de Artritis Reumatoide), REPANARC (Pan-American Registry of Early–Onset Arthritis) and BRASILIA, a Brazilian cohort [21-23]. For instance, the mean age at the disease onset in our cohort was 41 years, similar to that found in GLADAR (46 years), REPANARC (42 years) and BRASILIA (46 years); but younger than that described in European cohorts [19-25]. In addition, the proportion of women to men is higher in our cohort, as well as in the Latin American cohorts, compared to European and North American cohorts. When comparing pharmacologic treatments, MTX was the most commonly used DMARD and it was used in a similar frequency as in our study. Conversely, the exposure to TNF-α inhibitors was much lower in the Latin American cohorts compared to ours, which may reflect the disparities in terms of access to these medications. It has to be noted that a great diversity exists in terms of genetic and socioeconomic backgrounds and access to healthcare among different Latin American populations; hence, observations from these studies may not necessarily be extrapolated to other Hispanic populations.

Regarding the use of DMARDs, we found no difference between the study groups (early versus late treatment) in terms of specific initial or cumulative medications used suggesting that the beneficial effects of early therapy are based primarily on the institution of treatment within 6 months of symptom onset rather than the choice of therapy utilized. This concept has been shown in prior studies where early institution of DMARDs, regardless of specific drug selection, is associated with less radiographic progression and functional disability [20, 26-28].

We found no differences in the presence of traditional serologic markers or DAS28 scores between the early and late treatment groups. This apparent incongruence between serological markers and clinical response measures and the presence of surrogate markers of subclinical inflammation has been noticed before and it has been suggested that it could be related to the dissociation of inflammation and disease progression and its relation to persistent inflammatory state and the beginning of structural damage early in the disease course [29].

Regarding comorbid conditions, we found no difference between early and late treatment groups except for type 2 DM. The significantly higher prevalence of type 2 DM in the early treatment group is difficult to explain considering that these patients had similar health-related behaviors, frequency of overweight/obesity, and cumulative exposure to corticosteroids, all known risk factors for diabetes mellitus [30]. However, it must be noted that we calculated the cumulative presence of comorbidities even if they present before the diagnosis of RA. When we examined the percentage of incident cases of diabetes (after the onset of RA) we found no significant differences between early and late treatment groups. Previous studies have demonstrated that early RA treatment can positively impact cardiovascular risk by decreasing endothelial damage and reactivity [31, 32]. However, in our cohort there were no differences between the groups in terms of cardiovascular events. A plausible explanation for these findings could be related to the fact that the initial and cumulative treatment with DMARDs, including MTX, hydroxychloroquine and TNF-α inhibitors was similar in both groups. Studies have shown that the use of these medications as well as combination therapy can have a protective cardiac effect [33-37].

This study is not without limitations. First, it was an observational study of university and community-based rheumatology practices and thus may not be representative of the general population. In addition, no follow-up radiographic monitoring protocol was established posing a limitation in the evaluation of radiographic progression. This limitation could explain the lack of concordance between the higher frequency of deformities and replacement surgeries seen in the late treatment group and the lack of significant difference of radiographic damage between the early and late treatment groups. Finally, we did not evaluate the impact of early treatment in other important outcomes such as sustained clinical remission, work disability, and mortality. Nonetheless, our study presents clinically relevant data and outcomes in a cohort followed form a long-term of almost 15 years.

## CONCLUSION

Our data support the beneficial effects of early treatment in RA. Using the ACR definition of early RA as a time frame for early treatment, we found that RA patients who received conventional and/or biologic DMARDs within 6 months from the onset of symptoms attributable to RA had better long-term outcomes, having less physical damage and functional impairment than those who received late therapy. This time frame encourages its utilization for uniform data gathering and provides a sound reference when managing new patients with early RA that will benefit from prompt initiation of pharmacologic therapy, thus positively altering the impact of disease progression.

## Figures and Tables

**Table 1:** Demographic features and health-related behaviors in RA patients receiving early and late treatment with DMARDs (n=387).

**Features**	**Early treatment** **(n=121)**	**Late** **treatment** **(n=266)**	**p value**
Age at study visit, mean (SD) years	53.6 (14.0)	57.1 (13.8)	0.022
Gender, % female	86.8	87.2	0.905
Current alcohol consumption, %	6.6	4.2	0.300
Current cigarette smoking, %	7.4	6.0	0.598
Current exercise, %	20.2	15.7	0.277

RA: Rheumatoid Arthritis; DMARDs: Disease-Modifying Anti-rheumatic Drugs; SD: Standard Deviation.

**Table 2:** Clinical manifestations, disease activity and outcomes in RA patients receiving early and late treatment with DMARDs (n=387).

**Clinical Manifestations**	**Early treatment** **(n=121)**	**Late treatment** **(n=266)**	**p value**
Duration of RA, mean (SD) years	15.8 (91.0)	14.6 (10.9)	0.834
Joint deformities/contractures, %	35.5	55.3	<0.001
Radiographic evidence of joint damage, %	60.8	69.0	0.146
Positive RF and/or anti-CCP antibodies, %	67.6	75.9	0.222
Extra-articular manifestations, %	43.8	54.1	0.059
Ocular	29.8	37.6	0.134
Pulmonary	5.0	8.3	0.244
Cardiac	0.0	0.75	1.000
Neurologic	20.7	19.2	0.733
Subcutaneous nodules	19.0	29.7	0.027
Intra-articular steroid injections, %	40.0	54.5	0.008
Joint replacement surgery, %	9.2	22.6	0.002
RA exacerbations, mean (SD)	1.8 (2.8)	2.4 (3.1)	0.112
Hospitalizations, mean (SD)	0.1 (0.0)	0.2 (0.0)	0.150
DAS28, mean (SD)	3.5 (1.5)	3.7 (1.5)	0.357
Clinical remission (DAS28 < 2.6), %	24.8	22.6	0.629
Patient’s functional status, HAQ, mean (SD)	0.9 (0.7)	1.2 (0.8)	<0.001
Patient’s global assessment, mean (SD)	41.1 (28.1)	46.2 (30.0)	0.117
Physician’s global assessment, mean (SD)	17.1 (19.4)	22.0 (22.3)	0.041
Physician’s assessment of functional impairment, mean (SD)	16.3 (19.8)	25.1 (24.5)	<0.001
Physician’s assessment of physical damage, mean (SD)	14.3 (18.1)	24.1 (25.7)	<0.001

RA: Rheumatoid Arthritis; DMARDs: Disease-Modifying Anti-Rheumatic Drugs; SD: Standard Deviation; RF: Rheumatoid Factor; anti-CCP: Anti*-*Citrullinated Protein; DAS28: Disease Activity Score 28; HAQ: Health Assessment Questionnaire.

**Table 3:** Comorbid conditions in RA patients receiving early and late treatment with DMARDs (n=387).

**Comorbid Conditions**	**Early treatment** **(n=121), %**	**Late treatment** **(n=266), %**	**p value**
Arterial hypertension	52.1	57.5	0.317
Type 2 diabetes mellitus	20.6	12.8	0.046
Dyslipidemia	48.8	48.5	0.962
Overweight/obesity	65.8	65.5	0.952
Cardiovascular events	8.3	11.7	0.315
Cataracts	12.5	15.0	0.509
Glaucoma	5.8	4.5	0.591
Depression	11.6	14.3	0.468
Malignancy	4.1	4.5	0.866
Sjögren’s syndrome	11.6	7.5	0.192
Osteoarthritis	61.2	62.4	0.814
Osteoporosis	17.4	24.4	0.120

RA: Rheumatoid Arthritis; DMARDs: Disease-Modifying Anti-Rheumatic Drugs.

**Table 4:** Initial and cumulative pharmacologic treatments in RA patients receiving early and late treatment with DMARDs.

**Medications**	**Initial treatment**			**Cumulative treatment**		
	Early treatment (n=121) %	Late treatment (n=266) %	p value	Early treatment (n=121) %	Late treatment (n=266) %	p value
NSAIDs	52.9	59.0	0.259	84.3	86.8	0.503
Corticosteroids	50.4	48.9	0.779	73.3	79.0	0.224
Conventional DMARDs						
Methotrexate	57.8	63.0	0.334	83.5	86.5	0.438
Hydroxychloroquine	40.8	34.1	0.205	57.0	53.4	0.505
Sulfazalazine	5.8	4.9	0.730	15.7	16.5	0.836
Leflunomide	0.0	1.1	0.241	3.3	5.3	0.397
Azathioprine	0.0	1.1	0.240	2.5	4.1	0.415
Cyclophosphamide	0.0	0.0	---	0.8	0.8	0.938
Gold salts	4.1	2.7	0.450	7.4	8.7	0.689
Biologic agents						
TNF-α blockers	10.7	11.6	0.794	52.1	48.5	0.515
Abatacept	0.0	0.8	0.341	5.0	7.5	0.351
Rituximab	0.0	0.0	---	0.0	0.8	0.339
Tocilizumab	0.0	0.0	---	0.8	0.0	0.138
Small molecules						
Tofacitinib	0.0	0.0	---	0.0	0.38	0.499
Drug combinations						
NSAIDs + DMARDs	50.4	56.0	0.305	80.2	84.2	0.327
NSAIDs + biologics	4.1	3.8	0.860	44.6	41.7	0.593
Corticosteroids + DMARDs	47.1	47.0	0.983	67.8	75.6	0.109
Corticosteroids + biologics	5.0	3.4	0.457	38.8	41.4	0.641
DMARDs + biologics	5.0	4.2	0.714	47.1	44.0	0.567

RA: Rheumatoid Arthritis; NSAIDs: Nonsteroidal Anti-Inflammatory Drugs; DMARDs: Disease-Modifying Anti-rheumatic Drugs; TNF-α: Tumor Necrosis Factor-α

**Table 5:** Logistic regression analysis of the effect of early treatment on clinical manifestations in rheumatoid arthritis patients.

**Features**	**Un-Adjusted OR** **(95% CI)**	Adjusted OR* **(95% CI)**
Joint deformities	0.44 (0.28-0.70)	0.49 (0.31-0.78)
Subcutaneous nodules	0.56 (0.33-0.94)	0.61 (0.36-1.04)
Intra-articular steroid injections	0.56 (0.36-0.86)	0.61 (0.39-0.96)
Joint replacement surgery	0.35 (0.17-0.67)	0.37 (0.18-0.72)
Type 2 diabetes mellitus	1.77 (1.01-3.14)	2.13 (1.18-3.85)
Health assessment questionnaire	0.54 (0.35-0.83)	0.53 (0.34-0.83)
Physician’s global assessment	0.57 (0.36-0.87)	0.56 (0.36-0.88)
Physician’s assessment of functional impairment	0.52 (0.33-0.81)	0.54 (0.35-0.86)
Physician assessment of physical damage	0.55 (0.36-0.86)	0.58 (0.37-0.91)

OR: Odds Ratio; CI: Confidence Interval.

*Adjusted model includes age and sex.

## References

[1] Lee D.M., Weinblatt M.E. (2001). Rheumatoid arthritis.. Lancet.

[2] Pincus T., Callahan L.F., Sale W.G., Brooks A.L., Payne L.E., Vaughn W.K. (1984). Severe functional declines, work disability, and increased mortality in seventy-five rheumatoid arthritis patients studied over nine years.. Arthritis Rheum..

[3] Goekoop-Ruiterman Y.P., de Vries-Bouwstra J.K., Allaart C.F., van Zeben D., Kerstens P.J., Hazes J.M., Zwinderman A.H., Ronday H.K., Han K.H., Westedt M.L., Gerards A.H., van Groenendael J.H., Lems W.F., van Krugten M.V., Breedveld F.C., Dijkmans B.A. (2005). Clinical and radiographic outcomes of four different treatment strategies in patients with early rheumatoid arthritis (the BeSt study): a randomized, controlled trial.. Arthritis Rheum..

[4] Möttönen T., Hannonen P., Korpela M., Nissilä M., Kautiainen H., Ilonen J., Laasonen L., Kaipiainen-Seppänen O., Franzen P., Helve T., Koski J., Gripenberg-Gahmberg M., Myllykangas-Luosujärvi R., Leirisalo-Repo M., FIN-RACo Trial Group. FINnish Rheumatoid Arthritis Combination therapy (2002). Delay to institution of therapy and induction of remission using single-drug or combination-disease-modifying antirheumatic drug therapy in early rheumatoid arthritis.. Arthritis Rheum..

[5] Möttönen T.T., Hannonen P.J., Boers M. (1999). Combination DMARD therapy including corticosteroids in early rheumatoid arthritis.. Clin. Exp. Rheumatol..

[6] Landewé R.B., Boers M., Verhoeven A.C., Westhovens R., van de Laar M.A., Markusse H.M., van Denderen J.C., Westedt M.L., Peeters A.J., Dijkmans B.A., Jacobs P., Boonen A., van der Heijde D.M., van der Linden S. (2002). COBRA combination therapy in patients with early rheumatoid arthritis: long-term structural benefits of a brief intervention.. Arthritis Rheum..

[7] Lard L.R., Visser H., Speyer I., vander Horst-Bruinsma I.E., Zwinderman A.H., Breedveld F.C., Hazes J.M. (2001). Early versus delayed treatment in patients with recent-onset rheumatoid arthritis: comparison of two cohorts who received different treatment strategies.. Am. J. Med..

[8] Harris J.A., Bykerk V.P., Hitchon C.A., Keystone E.C., Thorne J.C., Boire G., Haraoui B., Hazlewood G., Bonner A.J., Pope J.E., CATCH Investigators (2013). Determining best practices in early rheumatoid arthritis by comparing differences in treatment at sites in the Canadian Early Arthritis Cohort.. J. Rheumatol..

[9] Breedveld F.C., Weisman M.H., Kavanaugh A.F., Cohen S.B., Pavelka K., van Vollenhoven R., Sharp J., Perez J.L., Spencer-Green G.T. (2006). The PREMIER study: A multicenter, randomized, double-blind clinical trial of combination therapy with adalimumab plus methotrexate versus methotrexate alone or adalimumab alone in patients with early, aggressive rheumatoid arthritis who had not had previous methotrexate treatment.. Arthritis Rheum..

[10] Emery P., Breedveld F.C., Hall S., Durez P., Chang D.J., Robertson D., Singh A., Pedersen R.D., Koenig A.S., Freundlich B. (2008). Comparison of methotrexate monotherapy with a combination of methotrexate and etanercept in active, early, moderate to severe rheumatoid arthritis (COMET): A randomised, double-blind, parallel treatment trial.. Lancet.

[11] Singh J.A., Saag K.G., Bridges S.L., Akl E.A., Bannuru R.R., Sullivan M.C., Vaysbrot E., McNaughton C., Osani M., Shmerling R.H., Curtis J.R., Furst D.E., Parks D., Kavanaugh A., O’Dell J., King C., Leong A., Matteson E.L., Schousboe J.T., Drevlow B., Ginsberg S., Grober J., St Clair E.W., Tindall E., Miller A.S., McAlindon T. (2016). 2015 American college of rheumatology guideline for the treatment of rheumatoid arthritis.. Arthritis Rheumatol..

[12] Santiago-Casas Y., González-Rivera T., Castro-Santana L., Ríos G., Martínez D., Rodríguez V., González-Alcover R., Mayor A.M., Vilá L.M. (2013). Impact of managed care health insurance system for indigent patients with rheumatoid arthritis in Puerto Rico.. Clin. Rheumatol..

[13] Villafrádez-Díaz M., Santiago-Casas Y., Nieves-Plaza M., Morales M., Rodríguez V., Ríos G., Martínez D., Vilá L.M. (2014). Association of the use of statins with disease activity and functional status in Puerto Ricans with rheumatoid arthritis.. P. R. Health Sci. J..

[14] Arnett F.C., Edworthy S.M., Bloch D.A., McShane D.J., Fries J.F., Cooper N.S., Healey L.A., Kaplan S.R., Liang M.H., Luthra H.S. (1988). The American Rheumatism Association 1987 revised criteria for the classification of rheumatoid arthritis.. Arthritis Rheum..

[15] Fransen J., van Riel P.L. (2005). The disease activity score and the EULAR response criteria.. Clin. Exp. Rheumatol..

[16] Hochberg M.C., Chang R.W., Dwosh I., Lindsey S., Pincus T., Wolfe F. (1992). The American College of Rheumatology 1991 revised criteria for the classification of global functional status in rheumatoid arthritis.. Arthritis Rheum..

[17] Anderson J.K., Zimmerman L., Caplan L., Michaud K. (2011). Measures of rheumatoid arthritis disease activity: Patient (PtGA) and Provider (PrGA) Global Assessment of Disease Activity, Disease Activity Score (DAS) and Disease Activity Score with 28-Joint Counts (DAS28), Simplified Disease Activity Index (SDAI), Clinical Disease Activity Index (CDAI), Patient Activity Score (PAS) and Patient Activity Score-II (PASII), Routine Assessment of Patient Index Data (RAPID), Rheumatoid Arthritis Disease Activity Index (RADAI) and Rheumatoid Arthritis Disease Activity Index-5 (RADAI-5), Chronic Arthritis Systemic Index (CASI), Patient-Based Disease Activity Score With ESR (PDAS1) and Patient-Based Disease Activity Score without ESR (PDAS2), and Mean Overall Index for Rheumatoid Arthritis (MOI-RA).. Arthritis Care Res. (Hoboken).

[18] Maska L., Anderson J., Michaud K. (2011). Measures of functional status and quality of life in rheumatoid arthritis: Health Assessment Questionnaire Disability Index (HAQ), Modified Health Assessment Questionnaire (MHAQ), Multidimensional Health Assessment Questionnaire (MDHAQ), Health Assessment Questionnaire II (HAQ-II), Improved Health Assessment Questionnaire (Improved HAQ), and Rheumatoid Arthritis Quality of Life (RAQoL).. Arthritis Care Res. (Hoboken).

[19] Combe B., Benessiano J., Berenbaum F., Cantagrel A., Daurès J.P., Dougados M., Fardellone P., Fautrel B., Flipo R.M., Goupille P., Guillemin F., Le Loet X., Logeart I., Mariette X., Meyer O., Ravaud P., Rincheval N., Saraux A., Schaeverbeke T., Sibilia J. (2007). The ESPOIR cohort: A ten-year follow-up of early arthritis in France: Methodology and baseline characteristics of the 813 included patients.. Joint Bone Spine.

[20] Combe B., Rincheval N., Benessiano J., Berenbaum F., Cantagrel A., Daurès J.P., Dougados M., Fardellone P., Fautrel B., Flipo R.M., Goupille P., Guillemin F., Le Loët X., Logeart I., Mariette X., Meyer O., Ravaud P., Saraux A., Schaeverbeke T., Sibilia J. (2013). Five-year favorable outcome of patients with early rheumatoid arthritis in the 2000s: Data from the ESPOIR cohort.. J. Rheumatol..

[21] Cardiel M.H., Pons-Estel B.A., Sacnun M.P., Wojdyla D., Saurit V., Marcos J.C., Pinto M.R., Cordeiro de Azevedo A.B., da Silveira I.G., Radominski S.C., Ximenes A.C., Massardo L., Ballesteros F., Rojas-Villarraga A., Oñate R.V., Hernandez M.P., Esquivel-Valerio J.A., García-De La Torre I., Khoury V.J., Millán A., Soriano E.R., GLADAR (2012). Treatment of early rheumatoid arthritis in a multinational inception cohort of Latin American patients: the GLADAR experience.. J. Clin. Rheumatol..

[22] Ramagli A., Corbacho I., Linhares F., de Abreu P., Teijeiro R., Garau M., Dapueto J. (2015). Characteristics of patients with early-onset arthritis in latin america: Description of the REPANARC cohort.. J. Clin. Rheumatol..

[23] da Mota L.M., Laurindo I.M., dos Santos Neto L.L. (2010). Demographic and clinical characteristics of a cohort of patients with early rheumatoid arthritis.. Rev. Bras. Reumatol..

[24] Marcos J., Waimann C., Dal Pra F., Hogrefe J., Retamozo S., Caeiro F., Casalla L., Benegas M., Rillo O., Spindler A., Berman H., Berman A., Secco A., García Salinas R., Catalán Pellet A., Ceccato F., Paira S., Marcos J.C., Maldonado Cocco J.A., Citera G., CONAART (Consorcio Argentino de Artritis Temprana–Argentine Consortium for Early Arthritis) (2011). General characteristics of an early arthritis cohort in Argentina.. Rheumatology (Oxford).

[25] van Nies J.A., Tsonaka R., Gaujoux-Viala C., Fautrel B., van der Helm-van Mil A.H. (2015). Evaluating relationships between symptom duration and persistence of rheumatoid arthritis: Does a window of opportunity exist? Results on the Leiden early arthritis clinic and ESPOIR cohorts.. Ann. Rheum. Dis..

[26] Bosello S., Fedele A.L., Peluso G., Gremese E., Tolusso B., Ferraccioli G. (2011). Very early rheumatoid arthritis is the major predictor of major outcomes: clinical ACR remission and radiographic non-progression.. Ann. Rheum. Dis..

[27] Lukas C., Combe B., Ravaud P., Sibilia J., Landew R., van der Heijde D. (2011). Favorable effect of very early disease-modifying antirheumatic drug treatment on radiographic progression in early inflammatory arthritis: Data from the Étude et Suivi des polyarthrites indifférenciées récentes (study and followup of early undifferentiated polyarthritis).. Arthritis Rheum..

[28] Kyburz D., Gabay C., Michel B.A., Finckh A., physicians of SCQM-RA (2011). The long-term impact of early treatment of rheumatoid arthritis on radiographic progression: a population-based cohort study.. Rheumatology (Oxford).

[29] Brown A.K., Conaghan P.G., Karim Z., Quinn M.A., Ikeda K., Peterfy C.G., Hensor E., Wakefield R.J., O’Connor P.J., Emery P. (2008). An explanation for the apparent dissociation between clinical remission and continued structural deterioration in rheumatoid arthritis.. Arthritis Rheum..

[30] Nguyen N.T., Nguyen X.M., Lane J., Wang P. (2011). Relationship between obesity and diabetes in a US adult population: Findings from the national health and nutrition examination survey, 1999-2006.. Obes. Surg..

[31] Chatterjee S., Sarkate P., Ghosh S., Biswas M., Ghosh A. (2013). Early, structured disease modifying anti-rheumatic drug (DMARD) therapy reduces cardiovascular risk in rheumatoid arthritis--a single centre study using non-biologic drugs.. J. Assoc. Physicians India.

[32] Kotani K., Miyamoto M., Ando H. (2017). The Effect of treatments for rheumatoid arthritis on endothelial dysfunction evaluated by flow-mediated vasodilation in patients with rheumatoid arthritis.. Curr. Vasc. Pharmacol..

[33] Westlake S.L., Colebatch A.N., Baird J., Kiely P., Quinn M., Choy E., Ostor A.J., Edwards C.J. (2010). The effect of methotrexate on cardiovascular disease in patients with rheumatoid arthritis: a systematic literature review.. Rheumatology (Oxford).

[34] Sharma TS, Wasko MC, Tang X (2016). Hydroxychloroquine wse is associated with decreased incident cardiovascular events in rheumatoid arthritis patients.. J Am Heart Assoc.

[35] Al-Aly Z., Pan H., Zeringue A., Xian H., McDonald J.R., El-Achkar T.M., Eisen S. (2011). Tumor necrosis factor-α blockade, cardiovascular outcomes, and survival in rheumatoid arthritis.. Transl. Res..

[36] Barnabe C., Martin B.J., Ghali W.A. (2011). Systematic review and meta-analysis: Anti-tumor necrosis factor α therapy and cardiovascular events in rheumatoid arthritis.. Arthritis Care Res. (Hoboken).

[37] Tikiz H., Arslan O., Pirildar T., Tikiz C., Bayindir P. (2010). The effect of anti-tumor necrosis factor (TNF)-alpha therapy with etanercept on endothelial functions in patients with rheumatoid arthritis.. Anadolu Kardiyol. Derg..

